# Mitochondrial Dysfunction in Dopaminergic Neurons Derived from Patients with LRRK2- and SNCA-Associated Genetic Forms of Parkinson’s Disease

**DOI:** 10.3390/cimb45100529

**Published:** 2023-10-17

**Authors:** Anna S. Vetchinova, Marina R. Kapkaeva, Mikhail V. Ivanov, Kristina A. Kutukova, Natalia M. Mudzhiri, Lydia E. Frumkina, Anatoly V. Brydun, Vladimir S. Sukhorukov, Sergey N. Illarioshkin

**Affiliations:** 1 Laboratory of Neurobiology and Tissue Engineering, Brain Science Institute, Research Center of Neurology, Moscow 125367, Russia; 2 Laboratory of Neuromorphology, Brain Science Institute, Research Center of Neurology, Moscow 125367, Russiamudzhiri@neurology.ru (N.M.M.);

**Keywords:** Parkinson’s disease, LRRK2, SCNA, alpha-synuclein, dardarin, induced pluripotent stem cells, dopaminergic neurons, transcriptomics, mitochondria

## Abstract

Parkinson’s disease (PD) is the second most common neurodegenerative disease. Some cases of PD may be caused by genetic factors, among which mutations in the LRRK2 and SNCA genes play an important role. To develop effective neuroprotective strategies for PD, it is important to diagnose the disease at the earliest stages of the neurodegenerative process. Therefore, the detection of diagnostic and prognostic markers of Parkinson’s disease (PD) is an urgent medical need. Advances in induced pluripotent stem cell (iPSC) culture technology provide new opportunities for the search for new biomarkers of PD and its modeling in vitro. In our work, we used a new technology for multiplex profiling of gene expression using barcoding on the Nanostring platform to assess the activity of mitochondrial genes on iPSC-derived cultures of dopaminergic neurons obtained from patients with LRRK2- and SNCA-associated genetic forms PD and a healthy donor. Electron microscopy revealed ultrastructural changes in mitochondria in both LRRK2 and SNCA mutant cells, whereas mitochondria in cells from a healthy donor were normal. In a culture with the SNCA gene mutation, the ratio of the area occupied by mitochondria to the total area of the cytoplasm was significantly lower than in the control and in the line with the LRRK2 gene mutation. Transcriptome analysis of 105 mitochondria proteome genes using the Nanostring platform revealed differences between the diseased and normal cells in the activity of genes involved in respiratory complex function, the tricarboxylic acid cycle, ATP production, mitochondria–endoplasmic reticulum interaction, mitophagy, regulation of calcium concentration, and mitochondrial DNA replication.

## 1. Introduction

Population aging has resulted in a notable rise in the number of patients suffering from neurodegenerative diseases in recent decades [1], requiring the introduction of new effective ways of treatment based on understanding of the molecular and cellular mechanisms of their pathogenesis. Parkinson’s disease (PD) is one of the most common age-related neurodegenerative disorders, primarily affecting dopaminergic neurons in the substantia nigra pars compacta (SNc). This results in severe impairment related to cognitive and motor function. Examining the neural substrates affected by PD is important for the broader understanding of this complex neurodegenerative disorder. Several hypotheses have been proposed to explain the progressive loss of neurons, including genetic predisposition, accumulation of abnormal proteins, mitochondrial changes, oxidative stress, chronic neuroinflammation, and aberrant neurogenesis [2,3,4]. The initial motor symptoms of PD manifest when approximately 50% of the dopaminergic neurons in the SNc have already degenerated, whereas the neurodegenerative process begins long before the clinical presentation [5]. Therefore, therapy may be ineffective even in early clinical stages, and the identification of early markers of the diagnostic and progression for PD is of utmost medical importance.

Inherited forms of PD have been associated with mutations in several genes, such as SNCA, PRKN, PINK1, LRRK2, DJ-1, VPS35, FBXO7, ATP13A2, and GBA [6]. 

The SNCA gene product, α-synuclein, was identified as the major component of Lewy bodies and Lewy neurites [7]. Duplications or triplications in the SNCA gene have been shown to contribute to autosomal dominant cases of PD with early onset, suggesting that overexpression of wild-type α-synuclein is sufficient for the pathogenesis of PD [8]. This small neuronal protein is detected in various regions of the brain, predominantly in the neocortex, hippocampus, and substantia nigra [9]. In normal conditions, the protein exists in a dynamic equilibrium between the states of unfolded monomers and organized α-helical tetramers [10]. Being in close proximity to vesicles in the presynaptic terminal, α-synuclein significantly influences synaptic transmission by regulating vesicle formation and neurotransmitter release [11]. In PD, mutant forms of α-synuclein tend to aggregate rather than disperse from synaptic boutons, leading to the deposition of synuclein-containing protofibrils [12]. The process of α-synuclein aggregation involves conformational changes that result in β-sheet-rich assemblies, facilitating its aggregation into oligomers, protofibrils, and insoluble fibrils, which ultimately accumulate in protein aggregates, known as Lewy bodies. The question of which forms of α-synuclein are cytotoxic remains open. Both oligomeric and fibrillar forms of α-synuclein, which form during the early stages of aggregation, are likely to be toxic and contribute to cellular demise in PD. Fibrils of α-synuclein appear to contribute to the propagation and progression of the disease, as they are found in the brains of patients with PD [13]. Misfolded α-synuclein can induce microglial activation, and activated microglia, in turn, can enhance aggregation and transcellular spreading of α-synuclein. A recent study suggests that microglial activation accompanied by the release of toxic cytokines, such as caspase-1 and calpains, plays a crucial role in disrupting the conformational folding of native α-synuclein and its subsequent spreading [14]. It has also been demonstrated that oligomeric forms of α-synuclein lead to mitochondrial damage and affect the production of reactive oxygen species [15]. 

Not all cases of PD are characterized by the presence of α-synuclein inclusions. Patients with an autosomal dominant form of PD carrying mutations in the LRRK2 gene, which encodes the protein dardarin, exhibit neuronal degeneration but do not exhibit Lewy bodies [16,17]. Dardarin is expressed at low levels in various regions of the brain, as well as in the lungs and other tissues, with the highest expression during the active formation of glutamatergic neurons. This large multidomain protein possesses protein kinase and GTPase activities and participates in various intracellular signaling pathways and cellular processes such as cytoskeletal dynamics, vesicle transport and endocytosis, autophagy, and mitochondrial metabolism. The two enzymatic roles of dardarin are associated with different regions of its molecule [18,19]. A number of amino acid substitutions in the coding region of the LRRK2 gene have been described in PD patients. For example, the G2019S mutation has been shown to alter the dynamics of mitochondria in glial cells, and brain lysates from rodent models of Parkinson’s disease demonstrate higher levels of pro-inflammatory factors TNF-α and Drp1, reflecting the role of LRRK2 in neuroinflammation [20].

Mitochondrial dynamics are being intensively investigated in PD. Despite the increased interest, the mechanisms underlying the initiation and regulation of these processes are still poorly understood. Both α-synuclein and dardarin are involved in the regulation of shared cellular processes, such as synaptic vesicle transport in neurons, cytoskeletal dynamics, protein degradation through the ubiquitin–proteasome system, autophagy, and mitochondrial function. It has been demonstrated that oligomeric forms of α-synuclein cause mitochondrial damage and impact the generation of reactive oxygen species [15], while an excess of dardarin exacerbates α-synuclein-induced mitochondrial swelling and superoxide production [21]. Although direct interaction between α-synuclein and dardarin has not been confirmed, the latter indirectly influences the aggregation of α-synuclein [22].

Modern technologies for obtaining and culturing induced pluripotent stem cells (iPSCs), derived from patients, have opened up new possibilities for studying the pathological mechanisms of neurodegenerative diseases and screening for personalized drug treatments. Neurons generated from PD-patient-derived iPSCs that carry various mutations represent highly informative in vitro PD models for elucidating the pathophysiological progression of the disease [23,24]. Importantly, iPSC-based models enable the detection of the earliest changes in neuronal morphology and functionality, allowing for the detection of the dynamics of pathological development at pre-symptomatic stages.

In our study, we used electron microscopy and a novel multiplex barcode-based gene expression profiling technology using the Nanostring platform to assess the activity of multiple genes involved in the development of neurodegenerative processes in iPSC cultures of dopaminergic neurons derived from PD patients and a healthy donor in order to identify early changes in neuronal function.

## 2. Materials and Methods

### 2.1. Materials

We used a CytoTune™ -iPS 2.0 Sendai Reprogramming Kit (Thermo Fisher Scientific, Waltham, MA, USA). The reprogramming vector includes the four Yamanaka factors Oct3/4, Sox2, Klf4, and c-Myc, shown to be sufficient for efficient reprogramming [25].

### 2.2. Cell Lines 

This work was carried out on the iPSC lines obtained from skin fibroblasts of a healthy donor and fibroblasts from two PD patients using the reprogramming kit. Cells from patients with PD carried the G2019S mutation in the LRRK2 gene or heterozygous duplication of 2–7 exons in the SNCA gene. The study complies with the Declaration of Helsinki and was approved by the Ethics Committee of the Research Center of Neurology (No. 11/12 of 12 September 2012). Written informed consent was obtained from each donor. All iPSC lines were cultured in mTeSR medium (STEMCELL Technologies Canada Inc., Vancouver, BC, Canada) on Matrigel-coated substrates. The differentiation of iPSCs into neural progenitor cells and further into differentiated neuronal cell cultures enriched with DA neurons was performed as previously described [26]. The pluripotency of the resulting lines was determined by assessing morphology, staining for alkaline phosphatase, staining with specific antibodies, and analysis of the expression of the Nanog, Oct4, Foxd3, and Hesx genes. 

### 2.3. Immunofluorescence and Transmission Electron Microscopy 

Two-dimensional neuronal cell cultures obtained from patients with PD (with a mutation in the LRRK2 and SNCA genes) and a healthy donor were plated on glass coverslips and used for immunofluorescence and TEM analysis. 

#### 2.3.1. Immunofluorescence 

Cells were fixed after 2 weeks of cultivation with 2% PFA for 30 min at room temperature and incubated with primary antibodies (mouse anti-b-III-tubulin, 1:500 (Biolegend Inc., San Diego, CA, USA); rabbit anti-tyrosine hydroxylase (TH), 1:500; rabbit anti-a-synuclein (total), 1:250 (Sigma-Aldrich, St. Louis, MO, USA); rabbit anti-a-synuclein (phospho S129), 1:250 (Abcam Inc., Cambridge, UK)) overnight at room temperature, followed by a 4 h incubation with appropriate secondary antibodies (CF448 donkey anti-mouse IgG, 1:250 (Sigma-Aldrich, St. Louis, MO, USA); Cy3 goat anti-rabbit IgG, 1:250 (Sigma-Aldrich, St. Louis, MO, USA)). DAPI was used to visualize cell nuclei. Antibody specificity was evaluated by performing immunohistochemical reactions with a negative and a positive control. As a negative control, immunohistochemical reaction with omission of the primary antibody was performed. As a positive control, rat and human tissue sections were used for immunohistochemical reactions: autopsy material of the substantia nigra taken from a patient with Lewy body disease for testing the antibodies against total and phosphorylated α-synuclein, and rat brain and intestine tissue sections for testing the anti-b-III-tubulin and anti-tyrosine hydroxylase antibodies. Cells were examined using a NikonEclipse NiU fluorescence microscope with a Nikon DS-Qi digital camera (Nikon Instruments Inc., Melville, NY, USA). Morphometric analysis was performed using the ImageJ software (version 1.54d) on pictures taken at ×40 magnification. The proportion of TH-positive neurons was assessed as TH-positive cell bodies/total DAPI-positive cell nuclei by counting. The mean intensity of fluorescent staining for phosphorylated α-synuclein was measured in the somata of neurons. Statistical analysis was carried out using the Statistica 6.0 software. Comparison of the studied parameters between groups was carried out using the Mann–Whitney test. Differences were considered statistically significant at *p* < 0.05.

#### 2.3.2. Transmission Electron Microscopy

Cells were fixed with 2% glutaraldehyde/0.1 M sodium phosphate buffer for 2 h at room temperature. The cells were then washed in 0.1 M sodium phosphate buffer, post-fixed in 1% OsO4 in 0.1 M sodium phosphate buffer for 2 h, dehydrated in graded series of ethanol, and embedded in Epon resin (Fluka Chemie GmbH, Buchs, Switzerland). Ultrathin sections of 50–70 nm were cut using an ultramicrotome LKB Bromma 8800 Ultratome III (Stockholm, Sweden), with a glass knife. The sections were collected on copper grids and contrasted with uranyl acetate and lead citrate. Rat brain tissue sections and a monolayer primary neural cell culture were used as control objects to validate the quality of samples obtained using this protocol. Imaging of the samples was done using a JEOL JEM-1011 transmission electron microscope (JEOL USA Inc., Peabody, MA, USA).

### 2.4. Isolation of RNA from a Culture of Neurons Obtained as a Result of Differentiation from iPSCs

Total RNA from neurons obtained from biopsy specimens from PD patients and a healthy donor was isolated using the Total RNA purification kit (Norgen Biotek Corp., Thorold, ON, Canada) according to the manufacturer’s recommendation. RNA was quantified using a Nanodrop 2000 (Thermo Fisher Scientific, Waltham, MA, USA). The RNA was either used immediately or stored at −80 °C during the experiments.

### 2.5. Analysis of Gene Expression Using nCounter Technology

Gene expression analysis was performed using the NanoString nCounter technology (NanoString Technologies, Seattle, WA, USA), which is based on direct barcoding of target RNA with fluorescent probes. In this study, a custom panel was used, which included 12 gene networks associated with mitochondrial function. The panel consisted of 112 target genes and 5 housekeeping genes, selected based on literature data on their involvement in mitochondrial processes. After hybridization of total RNA (100 ng) with the specific fluorescent probe set, samples were loaded onto the nCounter Analysis System (NanoString Technologies, Seattle, WA, USA) for data analysis following the manufacturer’s protocol. Data processing was conducted using the nSolver software package (version 4.0).

Primary data normalization was performed using 6 positive control probes and endogenous control genes included in the panel: β-actin (NM_001101.2), GAPDH (NM_002046.3), HPRT1 (NM_000194.1), RPL19 (NM_000981.3), and β-tubulin (NM_178014.2). The gene panel also included 8 negative control probes (non-complementary to any endogenous mRNA) to assess background noise. Data obtained using the nCounter system are expressed in units reflecting the concentration of target RNA molecules in the sample.

## 3. Results and Discussion 

### 3.1. Cell Reprogramming and Obtaining Cultures of Dopaminergic Neurons

**Figure 1:**
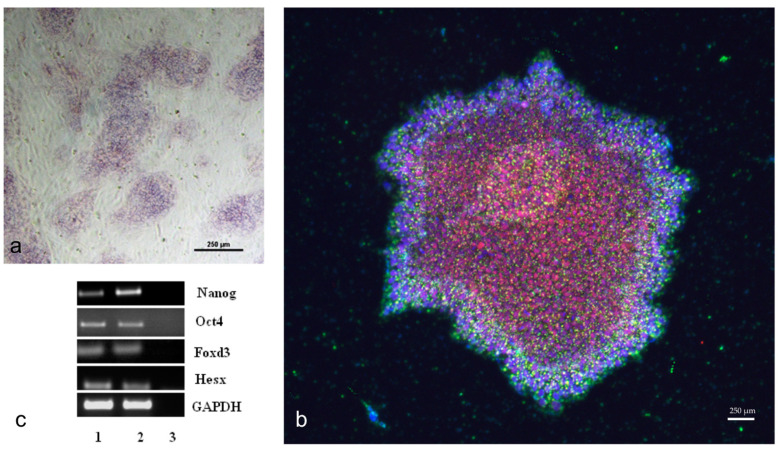
iPSC validation. (**a**)—Alkaline phosphatase staining of iPS colonies. (**b**)—Immunocytochemical staining of iPS cell clones from a patient with Parkinson’s disease for pluripotency markers: Sox2 (red), SSEA4 (green), DAPI (blue). (**c**)—Electrophoresis assessment of gene expression for pluripotency markers. 1, 2—iPSC, 3—negative control. Scale bar = 250 μm.

Patients with PD were studied using the MLPA method, which revealed heterozygous duplication of multiple exons (2–7) in the SNCA gene and the G2019S mutation in the LRRK2 gene. Skin biopsies were obtained from these patients and a control healthy donor. This study was approved by the local ethics committee of the Research Center of Neurology. All patients were informed about the study procedures and provided informed consent to participate. Homogenous cultures of primary skin fibroblasts were derived from the biopsy material of the patients and the donor. For fibroblast reprogramming, the Sendai virus was utilized, as this method does not result in integration of reprogramming factors and viral DNA into the genome. All necessary tests were performed on the induced pluripotent stem cells (iPSCs), obtained from PD patients and a healthy donor, according to international standards (Figure 1). These tests included validation of pluripotency marker expression, confirmation of normal karyotypes, and the ability to form embryoid bodies and derivatives of the three germ layers. The iPSCs from patients and the healthy individual were simultaneously differentiated into neural progenitors. The selection of iPSC lines was based on the results of the conducted tests. iPSC lines demonstrating a tendency towards preferential formation of neural derivatives in the spontaneous in vitro differentiation test were prioritized. Terminal differentiation into dopaminergic neurons was conducted in two stages.

### 3.2. Immunofluorescence Analysis of the Obtained Neuronal Cell Cultures

**Figure 2:**
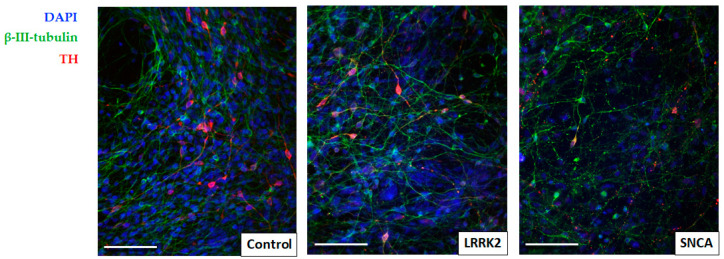
Immunofluorescence images showing representative colonies of iPSC-derived neurons in the control line, the line with the LRRK2 mutation, and the line with the SNCA mutation, stained for β-III-tubulin (green) and tyrosine hydroxylase (red), and counterstained with DAPI (blue) to visualize cell nuclei. Most of the cells in all three cultures are β-III-tubulin-positive neurons, and some of them are TH-positive dopaminergic neurons. Scale bar = 50 μm.

**Figure 3:**
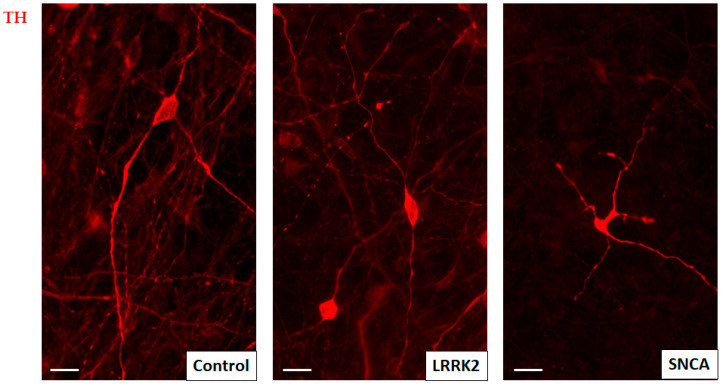
Representative morphology of TH-positive neurons in the control cell line, and the lines bearing mutations in the LRRK2 and SNCA genes. The majority of TH-positive neurons in both the control line and the line with LRRK2 mutation exhibit the same morphological features, such as spindle-shaped somata and two long processes arising from opposite sides of the soma. The TH-positive neurons in the culture with SNCA mutation generally have irregularly shaped somata and rather short varicose processes. Scale bar = 10 μm.

Most of the cells in all three cell cultures studied were considered neurons by expression of β-III-tubulin, some of them were also TH-positive (Figure 2). No difference in differentiation efficiency was detected across cell cultures studied, with an average of 15.7 ± 1.14% TH-positive neurons in the control culture, 14.4 ± 0.72% in the line with a mutation in the LRRK2 gene, and 14.8 ± 1.55% in the line with a mutation in the SNCA gene. TH-positive neurons had considerably small, fusiform cell bodies and long thin processes. TH-positive neurons with a mutation in the SNCA gene more often were not fusiform, but had an irregular, polygonal cell body shape, and their processes were short, branching, and with numerous bead-like extensions-varicosities (Figure 3). 

Total α-synuclein staining was detected in all cultures studied. The intensity of immunofluorescent staining of cells within the same colony was heterogeneous. Cells with high, medium, and low fluorescence intensity were visually identified. The latter numerically predominated, which corresponds to the literature data. In particular, Devine et al. (2011) showed a similar pattern of immunofluorescent staining for α-synuclein [27]. The observed heterogeneity of α-synuclein expression may be associated with the presence in the culture of different subtypes of neurons, or neurons at different stages of differentiation [28]. 

**Figure 4:**
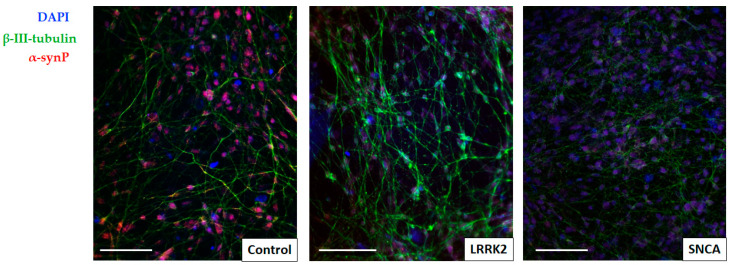
Immunofluorescence images showing expression of phosphorylated α-synuclein in iPSC-derived neuronal cultures. Phosphorylated α-synuclein (red) was visually detected in all cell cultures, in all β-III-tubulin neurons (green). Scale bar = 50 μm.

It is known that in patients with PD, as well as patients with dementia and Lewy bodies, up to 90% of α-synuclein (in Lewy bodies) is found in its Ser129-phosphorylated form, while only 4% of the phosphorylated protein is detected in a healthy brain [28,29]. We visually detected phospho-α-synuclein in all studied cell lines, in all cells. It showed a diffused staining pattern of the cell nuclei and and exhibited the form of small granules and punctated in the perikaryon and processes (Figure 4). The mean intensity of phospho-α-synuclein staining in culture cells with a mutation in the LRRK2 gene was significantly lower than in the control, while the cells of the line carrying the mutation in the SNCA gene did not differ from the control. Possibly, the lower intensity of immunofluorescence staining for phosphorylated α-synuclein in LRRK2 cells of the line with a mutation in the gene is associated with a high content of immature neurons in this cell culture [30].

### 3.3. Transmission Electron Microscopy Study of Neuronal Cultures

The neuronal culture from a healthy donor consisted mainly of small neurons, the nuclei of which had deep invaginations, one or several large nucleoli, often located eccentrically. The cytoplasm surrounded the nucleus with a thin rim; it was poor in organelles and filled mainly with polyribosomes and small dark mitochondria. Their processes were long and thin, with microtubules running in parallel to each other. In some cells, cisterns of the rough endoplasmic reticulum and the Golgi complex were present, as well as larger and lighter mitochondria compared with other cells, which is characteristic of more mature neurons. Also, cells with other morphologic features were present in the cell cultures. Such cells had large, light nuclei and a large volume of light cytoplasm filled with chaotically distributed cytoskeleton elements, and contained glycogen granules. Accumulations of small dark mitochondria were found in the perikaryon of these cells. We consider these cells to be immature neurons (neuronal progenitors).

**Figure 5:**
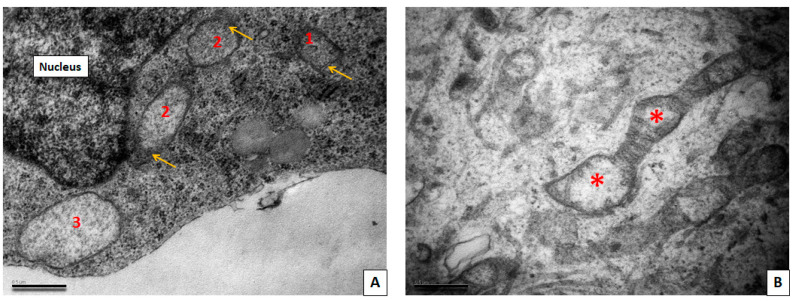
TEM images showing neurons with a mutation in LRKK2 gene. (**A**) Mitochodria showing different degrees of swelling and cristae loss (1–3). Remnants of cristae are marked with yellow arrows. (**B**) Mitochondria with centrally located zones of swelling (red asterisks). Scale bar = 0.5 μm.

Cells obtained from a patient with a mutation in the LRRK2 gene did not differ much in ultrastructure from the cells from a healthy patient, but this patient-derived cell culture tended to contain more cells with immature morphology. Some mitochondria in the cells with a mutation in the LRRK2 gene had very dark matrix, and others had lighter matrix and poorly developed cristae. There were also mitochondria with electron-light areas in the center, as well as mitochondria at different stages of loss of cristae and transformation into two-membrane light vacuoles (Figure 5). 

**Figure 6:**
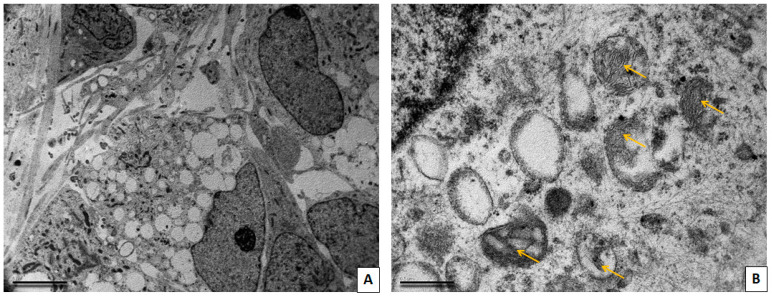
TEM images showing neurons with a mutation in the SNCA gene. (**A**) Low magnification. General view of cells with many rounded vacuoles in cytoplasm. Scale bar = 5 μm. (**B**) High magnification. Multiple vacuoles in perikarion; remnants of mitochondrial cristae are still present in some of them (yellow arrows). Scale bar = 0.5 μm.

Cells obtained from a patient with SNCA mutation showed numerous pathological changes (Figure 6). The cytoplasm of the majority of the cells was highly vacuolated. Vacuoles represented autophagolysosomes with content of various electron density, but predominantly they were transparent and swollen. Autophagic vacuoles were located both in the perinuclear part of the cytoplasm and also filled the cell processes. The remains of mitochondria could be seen inside of the autophagolysosomes. In the cell processes, there were enlarged areas in which the parallel course of microtubules was interrupted and where large autophagic vacuoles with different contents were located. These areas appear to correspond to bead-like varicosities seen on immunofluorescent images. A disruption of the cytoskeleton structure in these areas explains the appearance of bends and disturbances in the courses of processes, observed with light microscopy. Varicosities significantly impair the conduction of a nerve impulse along the axon and its transsynaptic transmission [31]. The appearance of axonal varicosities in neurons is characteristic of various neurological disorders, including Alzheimer’s disease, PD, and multiple sclerosis, and apparently reflects an increase in pathological changes in the axon during irreversible neurodegeneration [32]. In our culture with SNCA mutation, many cells contained a large nucleus and a large volume of cytoplasm which was poor in organelles, and this, in our opinion, corresponds to the ultrastructural features of immature neurons (neuronal progenitors). The cytoplasm of such cells is electron-lucent, there are few organelles of the protein-synthesizing apparatus in it, and chaotically arranged elements of the cytoskeleton predominated. Mitochondria were predominantly small and dark. Our results correlate with the existing research by Patt et al. [33], where, using Golgi impregnation, the authors have shown that dendrites of nigral neurons in the PD brain bear varicosities of different shape and size. Authors speculated that these varicosities could have Lewy pathology inclusions within them. In our immunofluorescence (IF) study, we also found the bead-like varicosities, especially in cells with SNCA mutation. These varicosities, however, did not contain alpha-synuclein aggregates. Our TEM assay also revealed varicose swellings of neuronal processes, but no electron-dense protein inclusions were found within them. These swellings were filled mostly with very large mitochondria or vacuoles with various contents. 

**Figure 7:**
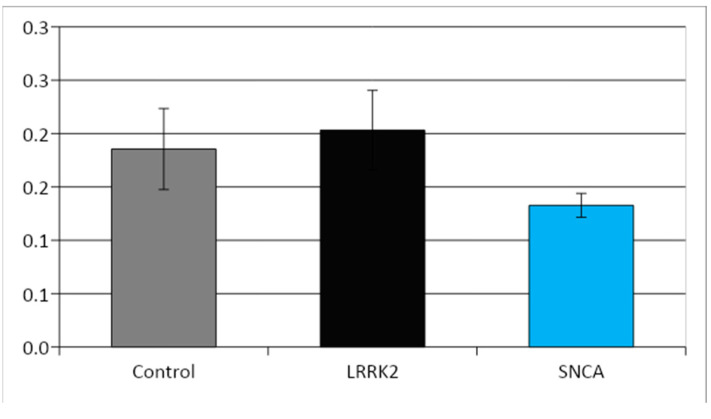
Mitochondrial area in the three neuronal lines, μm^2^ (Mean ± SEM).

**Figure 8:**
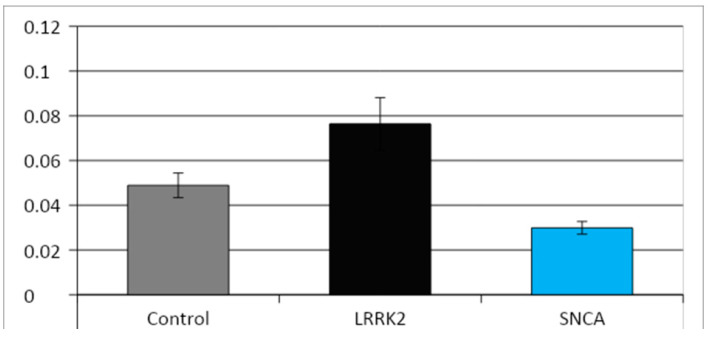
Area of mitochondria/area of cytoplasm ratio in the three neuronal lines (Mean ± SEM).

**Figure 9:**
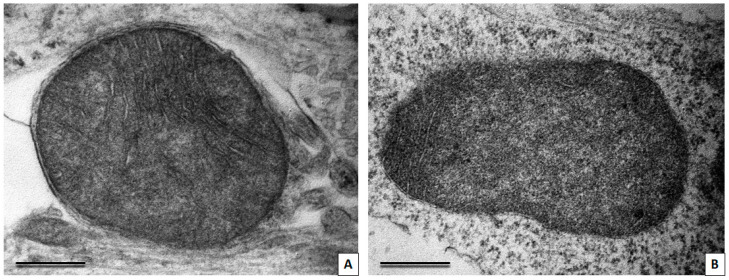
TEM images showing giant mitochondria in a neuron with LRRK2 mutation (**A**) and with SNCA mutation (**B**), showing similar features: dense matrix, irregular cristae, partial loss of cristae. Scale bar = 0.5 μm.

**Figure 10:**
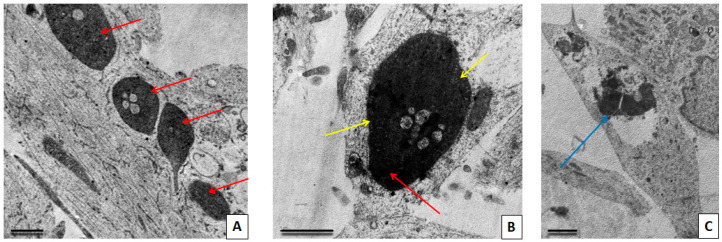
TEM images showing neurons with a mutation in SNCA gene. (**A**) Giant mitochondria within a process of a cell. Scale bar = 1 μm. (**B**) Giant (4.4 μm^2^) mitochondria with dark matrix, electron-dense inclusions (red arrows), and vacuoles. Remnants of cristae are visible along the periphery (yellow arrows). Scale bar = 1 μm. (**C**) Large autophagic vacuole in the process extension (blue arrow). Scale bar = 2 μm.

Morphometric study did not reveal statistically significant differences in the size of mitochondria in the studied cell lines (Figure 7). At the same time, in the cell culture with a mutation in the SNCA gene, the ratio of the area occupied by mitochondria to the total area of the cytoplasm was significantly lower than in the control and in the line with a mutation in the LRRK2 gene (Figure 8). In both cell lines with PD-associated mutations, individual giant mitochondria (with an area of 1.5–2.5 µm^2^, which is 10–20 times larger than the average size of mitochondria in the cells of these lines) were found, usually located inside the processes of the cells (Figure 9 and Figure 10). Such mitochondria had a dark matrix, altered cristae along the periphery, and vacuoles. The appearance of enlarged, giant mitochondria due to disruption of their normal dynamics can lead to difficulty in the transport of these organelles along the processes of neurons, suppression of synaptogenesis, as well as disruption of mitophagy processes, an increase in oxidative stress, and, ultimately, to cell death [34].

### 3.4. Gene Expression Analysis

**Figure 11:**
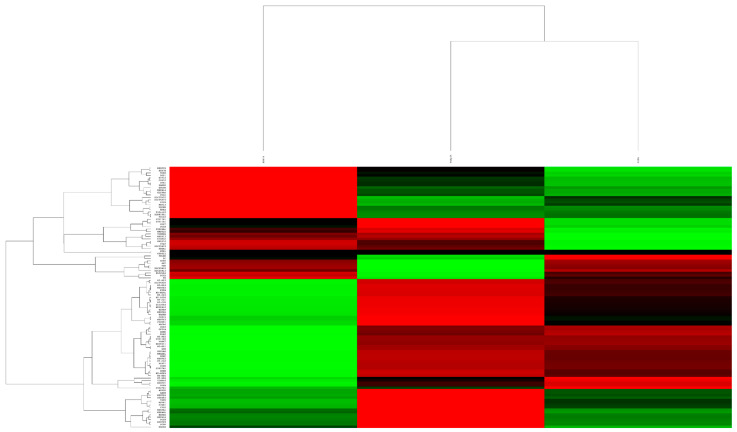
Heat map of changes in mitochondrial gene expression in neurons obtained from patients with PD and a conditionally healthy donor. Red: downregulated transcripts; green: activated transcripts; black: average expression of transcripts.

Next, we analyzed changes in the transcriptomic profiles of mitochondrial genes in three neuronal cultures derived from iPSCs using the Nanostring platform. The nCounter analysis system (Nanostring Technologies, Seattle, WA, USA) enables direct multiplex measurement of transcriptional activity of hundreds of genes and of translation levels of corresponding proteins, microRNA expression profiling, and gene copy number assessment (including within a single cell). The nCounter technology is based on direct digital detection of target sequences using fluorescent barcodes and aims to provide an absolute result: determining the quantitative content of the target product in a specific tissue or cell. The method is based on labeling targets with unique color-coded barcodes that are attached to target-specific probes, followed by their detection. By excluding the amplification step and the associated errors from the technological process, high levels of accuracy and sensitivity are demonstrated [35]. To achieve this, we measured the expression of a panel containing 12 gene networks associated with mitochondrial function in neuronal cultures differentiated from control- and patient-derived iPSCs (Figure 11).

Comparative analysis did not reveal statistically significant differences in the expression of COX4I2 (encoding mitochondrial enzyme isoform 2 of cytochrome C oxidase subunit 4) and PDX1 genes among all three cell lines. 

Our comparative analysis showed a decrease in the expression of COQ2, CYP11A1, CYP11B1, CYP24A1, DIABLO, HSPA13, MRPS16, MRPS22, PDHA1, SLC25A19, SPG7, SUOX, and TIMM8A genes in neurons derived from Parkinson’s disease patients compared with the expression of these genes in control neurons. The mitochondrial enzyme para-hydroxybenzoate-polyprenyl transferase functions in the final stages of CoQ (ubiquinone) biosynthesis, which is an electron carrier in the mitochondrial respiratory chain and a lipid-soluble antioxidant. This enzyme, part of the coenzyme Q10 pathway, catalyzes the prenylation of parahydroxybenzoate with an all-trans-polyisoprenyl group. Mutations in this gene cause coenzyme Q10 deficiency and mitochondrial encephalomyopathy. The products of CYP11A1, CYP11B1, and CYP24A1 genes belong to the cytochrome P450 superfamily. Enzymes in this group play important roles in the metabolism of steroids, bile acids, unsaturated fatty acids, phenolic metabolites, and the neutralisation of xenobiotics. Disruptions in the CYP24A1 gene can lead to increased calcium absorption. Mitochondria, in turn, are major regulators of calcium signaling and are capable of locally regulating cytosolic calcium concentration in different parts of the neuron. Local changes in calcium concentration and mitochondrial transport systems are involved in the pathogenesis of certain neurodegenerative diseases, including Parkinson’s disease [36]. The DIABLO gene encodes a protein that binds to the inhibitor of apoptosis protein (IAP). This mitochondrial protein enters the cytosol when cells undergo apoptosis and allows the activation of caspases by binding to apoptosis inhibitor proteins. Reduction in the expression of the encoded protein decreases the sensitivity of cells to apoptosis. The protein encoded by HSPA1 belongs to the heat shock protein 70 family and is associated with microsomes. Members of this protein family play a role in the processing of cytosolic and secretory proteins, as well as in the removal of denatured or incorrectly folded proteins. Mitochondrial ribosomal proteins encoded by MRPS16 and MRPS22 genes are involved in protein synthesis in mitochondria. The alpha subunit 1 of pyruvate dehydrogenase E1, the product of the PDHA1 gene, is involved in the conversion of pyruvate to acetyl-CoA and CO2, thereby linking the glycolytic pathway to the tricarboxylic acid cycle.

The mitochondrial protein encoded by the SLC25A19 gene transports a molecule called thiamin pyrophosphate into mitochondria. Thiamin pyrophosphate is involved in the functioning of a group of mitochondrial enzymes called the alpha-ketoglutarate dehydrogenase complex. This complex acts on a compound called alpha-ketoglutaric acid as part of the important series of reactions known as the citric acid cycle or Krebs cycle. The transport of thiamin pyrophosphate into mitochondria is believed to be crucial for brain development. The SPG7 gene encodes a mitochondrial metalloprotease, which is a member of the AAA family. Members of this family of proteins have a common ATPase domain and play a role in various cellular processes, including membrane transport, intracellular mobility, organelle biogenesis, protein folding, and proteolysis. The enzyme encoded by the SUOX gene catalyzes the oxidation of sulfite to sulfate, which is the final step in the oxidative degradation of sulfur-containing amino acids such as cysteine and methionine. Deficiency of sulfite oxidase leads to neurological disorders. The mitochondrial inner membrane translocase subunit TIMM8A is involved in the import and insertion of hydrophobic membrane proteins from the cytoplasm into the inner membrane of mitochondria. Proteins of the TOM multiprotein complex on the cytosolic surface of the outer mitochondrial membrane recognize the targeting signals of imported proteins. TIMM8A acts as a chaperone-like protein, protecting hydrophobic precursors from aggregation and guiding them through the intermembrane space of mitochondria. TIMM8A is considered an essential component for normal neurological development [37]. However, compared with neurons from Parkinson’s disease patients, the level of expression of CYP27B1, which encodes 1-alpha-hydroxylase (a cytochrome P450 enzyme), and PC, which encodes pyruvate carboxylase (an enzyme involved in gluconeogenesis, lipogenesis, insulin secretion, and glutamate neurotransmitter synthesis), in neurons from the healthy donor was slightly lower. 

Neurons derived from the patient with a mutation in the SNCA gene showed increased expression for 44 genes and decreased expression for 21 genes compared with control neurons and neurons with a mutation in the LRRK2 gene. For neurons derived from the PD patient with the G2019S mutation in the LRRK2 gene, we observed increased expression for 9 genes and decreased expression for 16 genes compared with control neurons and neurons with a mutation in the SNCA gene. The data are presented in Table 1.

A distinctive feature of midbrain dopaminergic neurons in PD patients is the impairment of respiratory chain enzyme function [38]. The five multisubunit enzyme complexes of the respiratory chain, embedded in the inner mitochondrial membrane and denoted by Roman numerals I-V, carry out oxidative phosphorylation (OXPHOS), which serves as the primary cellular energy source. These complexes comprise a total of up to 85 different subunits. Substrates provide reducing equivalents to complexes I (NADH-CoQ reductase) and II (succinate-CoQ reductase), and electrons are then transferred through the respiratory chain via ubiquinone and cytochrome C to complexes III (ubiquinol-cytochrome C reductase) and IV (cytochrome C oxidase). The electrochemical gradient is maintained by the pumping of protons across the inner mitochondrial membrane into the intermembrane space. The energy generated by the reverse translocation of protons into the matrix is utilized for ATP synthesis catalyzed by complex V (ATP synthase) [39]. Besides their energetic function, mitochondria also play a key role in various metabolic processes such as amino acid biosynthesis, fatty acid oxidation, steroid metabolism, calcium homeostasis, and the elimination of free radicals [40]. Through our study conducted on the Nanostring platform of neurons with a mutation in the *SNCA* gene, we found an increase in gene expression levels in genes encoding proteins involved in OXPHOS systems; protein import, sorting, and assembly; protein and lipid metabolism; and proteins capable of regulating the expression of other genes associated with oxidative stress, apoptosis, and neurotoxicity.

In neurons, the expression of the hypoxia-inducible transcription factor 1 (*HIF-1*) gene is relatively high. The HIF-1 transcription factor consists of a heterodimer composed of a constitutively expressed subunit, HIF-1β, and an oxygen-regulated subunit, HIF-1α, and responds to reduced oxygen availability by coordinating the expression of genes involved in adaptation to a hypoxic environment. Under normoxic conditions, HIF-1α rapidly degrades; however, under hypoxic conditions, the expression of HIF-1α stabilizes, allowing its nuclear translocation and formation of a heterodimer with HIF-1β [41]. Increased levels of HIF-1α lead to the upregulation of genes that promote cellular adaptation to hypoxia and stimulate erythropoiesis (erythropoietin genes), angiogenesis (vascular endothelial growth factor gene, *VEGF*), and glycolysis enzymes (aldolase, lactate dehydrogenase, phosphofructokinase, pyruvate kinase genes) [42]. The HIF factor influences the expression of tyrosine hydroxylase, a rate-limiting enzyme involved in the synthesis of catecholamines, specifically, the conversion of L-tyrosine into the precursor of dopamine. Hypoxia affects the expression of several transcripts encoding structural components of the mitochondria–endoplasmic reticulum contact sites, including genes encoding mitofusin. Mitofusin Mfn2 plays an essential role in mitochondrial fusion and is possibly a key regulator of the contacts between mitochondria and the endoplasmic reticulum [43]. Furthermore, Mfn2 is necessary for axonal transport of mitochondria through interaction with the kinesin motor protein complex and is a key participant in initiating PINK1-Parkin-induced mitophagy in dopaminergic neurons. It is important to note that the inactivation of Mfn2 impacts the organization of the inner mitochondrial membrane, which undergoes dynamic changes. The fusion of inner mitochondrial membranes involves the dynamin-like GTPase OPA1, which is also important for maintaining normal cristae structure and regulating cytochrome-C-induced apoptosis [44,45]. The reduction in the expression of HIF-1α, Mfn2, and OPA1 in neurons of the patient with a mutation in the *SNCA* gene may disrupt the integrity of mitochondrial membranes and result in the release of cytochrome C from the intermembrane space into the cytosol. 

The DNA polymerase gamma, encoded by the *POLG* gene, is involved in mitochondrial DNA replication and repair. In the patient with a mutation in the *SNCA* gene, decreased expression of both *POLG1* and *POLG2* is observed.

The product of the *TUFM* gene, the mitochondrial translation elongation factor, plays multiple roles in regulating mitochondrial components of the electron transport chain, inducing autophagy through the recruitment of the ATG12-5-16L1 complex, and inhibiting inflammation by binding to NLRX1, thus performing a crucial metabolic function [46]. In neurons of patients with PD, its expression level is reduced. 

The high expression level of a serine/threonine protein kinase, which is recruited and activated by double-strand DNA breaks, in neurons of patients with a mutation in the *LRRK2* gene, may be due to the interaction of the mutant guanosine triphosphate-dependent kinase with lamin B1 protein, associated with the inner nuclear membrane and involved in nuclear skeleton formation. This pathological interaction contributes to chromatin instability and disruption of the cell cycle [47]. ATM plays a crucial role in cell cycle arrest following DNA damage, particularly after double-strand breaks. Furthermore, in neurons of patients with mutant dardarin, increased expression of genes encoding enzymes of the Krebs cycle, frataxin, the alpha subunit of the heterodimeric mitochondrial enzyme propionyl-CoA carboxylase, the major catalytic subunit of succinate-ubiquinone oxidoreductase in the mitochondrial respiratory chain, proteins involved in the exchange of aspartate for a glutamate and a proton across the inner mitochondrial membrane, a transporter protein of phosphate ions across the inner membrane of mitochondria, trimethyllysine dioxygenase, the first enzyme in the biosynthesis pathway of carnitine, was observed. Carnitine plays a critical role in transporting activated fatty acids across the inner mitochondrial membrane.

In normal conditions, the serine/threonine kinase PINK1 acts as a sensor of mitochondrial damage and serves as a quality control element protecting neurons from oxidative stress. Mitophagy is stimulated when PINK1 accumulates in its active form on the outer mitochondrial membrane. The decreased expression of the *PINK1* gene observed in neurons of the patient with a mutation in the *LRRK2* gene may ultimately contribute to the accumulation of damaged mitochondria. The reduction in the expression level of *HSPA1A, HSPA4L, HSPA6,* and *HSPB1* may also impact neuronal survival during neurodegeneration. Through our study was conducted on the Nanostring platform, we identified a decrease in the expression level of genes encoding proteins involved in protein sorting, protein assembly, and nucleotide metabolism in neurons with a mutation in the *LRRK2* gene.

## 4. Conclusions

Mitochondria play a crucial role in generating and regulating cellular bioenergetics. Through their interaction with numerous signaling pathways, they contribute to neuronal development, plasticity, and differentiation, and play a central role in neuronal survival and death [48]. In our study, we identified and confirmed significant mitochondrial defects at the ultrastructural level and by analysing the expression changes of genes carrying information about the proteins of these organelles. Electron microscopy revealed pathologic changes in the neurons of a patient with Parkinson’s disease caused by a mutation in the *SNCA* gene. We observed larger mitochondria with altered cristae around the periphery and vacuoles. The presence of these enlarged, giant mitochondria resulting from the disruption of their normal dynamics can impede the transport of these organelles along neuronal processes, suppress synaptogenesis, impair mitophagy processes, increase oxidative stress, and ultimately lead to cellular death.

Through comparative analysis of the expression profiles of 105 genes in neurons derived from PD patients and a healthy donor, we were able to identify differences in the expression of genes involved in function of respiratory complexes, the tricarboxylic acid cycle, ATP production, mitochondria–endoplasmic reticulum interaction, mitophagy, regulation of calcium concentration, and mitochondrial DNA replication. 

It is important to note that various mitochondrial abnormalities at the structural and transcriptomic levels have been detected in two distinct mutations (*SNCA* and *LRRK2* genes) that are responsible for the development of PD. Nonetheless, it is evident that the mechanisms of damage in these cases were dissimilar. Thus, specifically, the reduction in mitochondrial fusion activity due to the *SNCA* gene mutation, which appears to account for the decline in the overall mitochondria area in neurons, has greater consequences for the fate of these organelles compared with the other mutation analyzed. Our findings provide a deeper and more detailed understanding of the well-established link between mitochondrial dysfunction and pathogenesis of PD. Our study suggests that pathologic changes occur at the earliest stages of neurogenesis. The results show possible directions of future investigations in patients with PD-associated mutations, while the knowledge of the general principles of mitochondrial disorders opens the field for possible early intervention aimed at delaying (at least) or halting the onset of clinical manifestations.

## Figures and Tables

**Table 1:** Gene networks associated with the work of the mitochondrial apparatus in cultures of neurons differentiated from the control patient and patients with PD.

Gene Expression Level	Control Neurons	Neurons with LRRK2 Mutation	Neurons with SNCA Mutation
	Gene Name		
Increased gene expression	*COQ2, CYP11A1, CYP11B1, CYP24A1, DIABLO, HSPA13, MRPS16, MRPS22, PDHA1, SLC25A19, SPG7, SUOX, TIMM8A*	*AMT, FH, FXN, PCCA, SDHA, SLC25A12, SLC25A13, SLC25A3, TMLHE*	*ABCB6, ACAT1, COX15, COX6B1, CPT1A, CYP11B2, CYP27A1, DLAT, ETFA, GSR, HADHB, HSPA9, MT-ATP6, MT-ATP8, MT-CO1, MT-CO2, MT-CO3, MT-CYB, MT-ND1, MT-ND2, MT-ND3, MT-ND4, MT-ND4L, MT-ND5, MT-ND6, NDUFA10, NDUFA11, NDUFB3, NDUFS2, NDUFS3, NDUFS6, NDUFV1, PARK7, PDHB, PDHX, RNASEL, SDHB, SDHC, SDHD, SLC25A20, SLC25A4, SOD2 SUCLA2, TIMM44*
Decreased gene expression	*CYP27B1, PC*	*ATP5E, ATPAF2, CYCS, GATM, GCDH, HADHA, HSPA1A, HSPA4L, HSPA6, HSPB1, NDUFA1, NDUFB9, NDUFS4, PCCB, PINK1, TSFM*	*ALDH18A1, ATP7B, C10orf2, COX10, DGUOK, HIF1A, HSPA14, MFN2, MRPL3, NDUFV2, OPA1, PDP1, POLG, POLG2, PPOX, SLC9A6, SARDH, SLC25A15, SLC25A22, TUFM, UQCRB*

## Data Availability

The data presented in this study are available within this paper.
